# BCL-2 Multi-Strain Probiotics for Immunomodulation In Vitro and In Vivo Alleviation of Atopic Dermatitis

**DOI:** 10.3390/microorganisms13081950

**Published:** 2025-08-21

**Authors:** MinKyung Sung, Seongrok Sim, Ahyoung Lim, Jin Seok Moon, JongIk Jeon, Keon Heo, Woongkwon Kwak, Myeong Soo Park, Jungki Kwak, EunYoung Park, Seokmin Yoon

**Affiliations:** 1 Lotte R&D Center, Seoul 07594, Republic of Korea; minkyung.sung@lotte.net (M.S.); ahyounglim@lotte.net (A.L.);; 2 Department of Biotechnology, College of Life Sciences and Biotechnology, Korea University, Seoul 02841, Republic of Korea; 3 Research Center, BIFIDO Co., Ltd., Hongcheon 25117, Republic of Korea; tjdfhr0108@bifido.com (S.S.); jsmoon@bifido.com (J.S.M.);

**Keywords:** atopic dermatitis, *Lactiplantibacillus plantarum*, *Bifidobacterium longum*, immune modulation, inflammatory cytokines, hypersensitivity, pro-inflammatory mediators, histopathology, mice

## Abstract

Atopic dermatitis (AD) is a chronic inflammatory disorder with immune imbalance, including elevated IgE levels and mast cell activation mediated by Th2 cytokines, leading to allergic inflammation and impaired skin barrier function. Current treatment limitations highlight the need for safer and more effective AD alternatives. We aimed to evaluate the therapeutic effects of multi-strain probiotics, BCL-2 (comprising *Lactiplantibacillus plantarum* LRCC5264 and *Bifidobacterium longum* RAPO), in alleviating AD clinical signs and elucidate its underlying immunomodulatory mechanisms. In vitro, BCL-2 treatment significantly reduced IL-4 secretion in RBL-2H3 cells, with higher inhibitory effects than single-strain treatment. In vivo, BCL-2 (10^6^–10^8^ CFU/day) was orally administered for 28 days to AD-induced Nc/Nga mice. BCL-2 treatment improved the clinical signs and histopathological features of AD, including epidermal hypertrophy, hyperkeratosis, and mast cell infiltration (*p* < 0.05). It also reduced neutrophil and eosinophil counts and modulated cytokine and chemokine profiles, notably decreasing IL-17, IL-5, IL-6, TNF-α, IL-1β, TARC, and eotaxin, while increasing IL-10, IFN-γ, and IL-12 (*p* < 0.05). Among the tested concentrations, 10^7^ CFU exhibited the most effective immune modulation with no adverse effects on body weight. These findings demonstrate the therapeutic potential of BCL-2 in AD; however, further studies are required to validate its clinical relevance.

## 1. Introduction

Atopic dermatitis (AD) is a chronic inflammatory skin disorder characterized by immune dysregulation, particularly an imbalance between Th1 and Th2 responses [1]. In the acute phase of AD, decreased IFN-γ production by Th1 cells is insufficient to suppress Th2-mediated responses, contributing to allergic inflammation, elevated IgE levels, and skin barrier dysfunction [1,2]. Th17 cells secrete IL-17, which compromises barrier integrity by disrupting tight junctions and promotes neutrophilic inflammation [3]. Th22 cells release IL-22, leading to keratinocyte hyperproliferation and epidermal thickening. These immune pathways are involved in the exacerbation of cutaneous inflammation and barrier impairment in AD [4]. Therefore, the effective modulation of these immune responses is a key goal in the treatment of AD [2,4].

Current therapeutic approaches for AD primarily involve antibody-based agents and anti-inflammatory drugs, including topical corticosteroids and calcineurin inhibitors administered to lesional skin, as well as systemic corticosteroids, which are reserved for severe or refractory cases [4,5]. Omalizumab, a monoclonal antibody, neutralizes IgE and suppresses mast cell activation, demonstrating its significant efficacy [5]. However, its efficacy is dose-dependent, and individuals with markedly elevated IgE levels may necessitate higher dosing [5]. Fixed-dose regimens may be insufficient to effectively neutralize circulating IgE, thereby limiting therapeutic outcomes due to persistent FcεRI-mediated mast cell activation [6]. Steroid-based anti-inflammatory therapies offer short-term symptom relief but are unsuitable for long-term management because of the risk of adverse effects [7]. These limitations have prompted growing interest in alternative therapies that provide safer and more sustainable immune modulation [8].

Probiotics, particularly *Lactobacillus* and *Bifidobacterium* species, have emerged as promising alternatives for treating AD [8,9]. These live microorganisms confer health benefits, such as improved gut health, immune regulation, and attenuation of pathological inflammation involved in various diseases [8]. In contrast, prebiotics are non-digestible substrates that selectively stimulate the growth of beneficial bacteria, while synbiotics refer to combinations of probiotics and prebiotics that act synergistically to enhance host health [9,10,11]. In the context of AD, probiotics have been shown to alleviate clinical signs including pruritus, erythema, xerosis, and lichenification by promoting Th1 and Treg responses, thereby reducing IgE levels and suppressing mast cell degranulation [12,13]. Notably, preclinical studies report enhanced suppression of mast cell degranulation when probiotics are co-administered with IgE-targeting agents, improving allergic symptom control without escalating antibody therapy doses [14]. Furthermore, probiotics are considered safe, with a long history of use and minimal adverse effects, supporting their therapeutic potential in AD.

In this study, we aimed to evaluate the alleviation of clinical signs and immune modulation by the multi-strain probiotic formulation BCL-2, composed of *Lactiplantibacillus plantarum* LRCC5264 (LP-5264) and *Bifidobacterium longum* RAPO (BL-RAPO), in a mouse model of AD. Furthermore, we examined immunological and histopathological parameters associated with these effects, highlighting that the novel BCL-2 formulation could represent a potential therapeutic alternative.

## 2. Materials and Methods

### 2.1. Bacterial Strains and Preparation

The probiotic strains LP-5264 and BL-RAPO were provided by Lotte R&D Center (Magok-juang-ro, Seoul, Republic of Korea) and Bifido Co., Ltd. (Nonggongdanji-gil, Hongcheon, Republic of Korea), respectively. In a previous study, LP-5264 was isolated from traditionally fermented kimchi, where it exhibited the highest survival under simulated gastrointestinal conditions and demonstrated superior anti-inflammatory activity by reducing LPS-induced IL-1β and TNF-α expression in RAW 264.7 cells, supporting its selection for the present study. The strain was deposited in the Korean Culture Center of Microorganisms (KCCM 13295P) and is described in the Korean Patent Application No. 10-2022-0190902. BL-RAPO was previously isolated by BIFIDO Co., Ltd. from fecal samples of healthy individuals and identified as a *Bifidobacterium* strain via 16S rRNA sequencing [15]. It was selected for its strong IL-17 inhibitory activity in vitro and deposited in the Korean Collection for Type Cultures (KCTC 13773BP).

Both strains were cultured in MRS broth under anaerobic conditions, aliquoted, and stored at −80 °C. For animal studies, large-scale fermentation was performed using a sterilized medium under controlled conditions. Cells in the exponential phase were harvested, freeze-dried, and quantified by serial dilution on MRS agar. All procedures were repeated in triplicate to ensure process consistency. The lyophilized strains were blended at a 1:1 ratio to produce the BCL-2 formulation, which was subsequently evaluated through both in vitro and in vivo experiments. In vitro, the mRNA expression levels of inflammatory cytokines were measured in RBL-2H3 cells. In vivo, its effects were assessed in a murine model of AD over a total period of 6 weeks, comprising 2 weeks of AD induction followed by 4 weeks of BCL-2 administration, during which clinical signs, serum cytokines, and histopathological alterations were evaluated.

### 2.2. In Vitro Cell Culture and Cytokine Analysis

Cytokine analysis experiments were conducted with reference to the methods described by Kim et al., with minor modifications [16]. RBL-2H3 rat basophilic leukemia mast cells were obtained from the American Type Culture Collection (ATCC, Manassas, VA, USA). The cells were maintained in Eagle’s Minimum Essential Medium (EMEM) supplemented with 15% fetal bovine serum, 1% penicillin/streptomycin, and 1 mM sodium pyruvate at 37 °C in a humidified incubator with 5% CO_2_. For cytokine assays, cells were seeded at a density of 5 × 10^5^ cells/mL in 48-well plates and sensitized overnight with 100 ng/mL dinitrophenyl-specific IgE (DNP-IgE). After washing twice with Siraganian buffer (containing 119 mmol/L NaCl, 5 mmol/L KCl, 0.4 mmol/L MgCl_2_, 25 mmol/L PIPES, 40 mmol/L NaOH, pH 7.2), cells were treated with LP-5264 and/or BL-RAPO at concentrations of 1.0 × 10^5^, 1.0 × 10^6^, or 1.0 × 10^7^ CFU/mL for 1 h. The cells were then stimulated with 100 ng/mL dinitrophenyl–bovine serum albumin (DNP-BSA) for 2 h. Culture supernatants were collected and analyzed for inflammatory cytokines using ELISA kits (R&D Systems, Minneapolis, MN, USA), following the manufacturer’s instructions.

### 2.3. Animals and Experimental Design

A total of 52 male Nc/Nga mice (5 weeks old) were purchased from Japan SLC, Inc. (Shizuoka, Japan) through the JoongAng Experimental Animal Center (Seoul, Republic of Korea). Mice were housed in polycarbonate cages (170 W × 235 L × 125 H mm), up to four per cage, under controlled conditions (temperature: 23 ± 3 °C, relative humidity: 55 ± 15%, ventilation: 10–20 air exchanges per h, 12 h light/dark cycle, and illumination: 150–300 lux) [15,16]. All mice were subjected to a 7-day acclimation period, during which general health, nutritional status, and clinical signs of disease were assessed. Based on these evaluations, 42 mice were selected for inclusion in the study, with no further exclusions thereafter.

After a 7-day acclimation period, mice were randomly assigned to six groups (*n* = 7 per group) [17]: G1, healthy control (no AD induction, no treatment); G2, negative control (AD-induced, no treatment); G3, positive control (AD-induced, treated with dexamethasone, 2 mg/kg); G4–G6, AD-induced and treated with BCL-2 at 10^6^, 10^7^, and 10^8^ CFU/day, respectively. AD was induced immediately following acclimation using Biostir^®^ AD (Biostir Inc., Osaka, Japan) over a 6-week period, with topical application performed twice weekly (total of 12 times). Initially, the ears and dorsal neck were shaved and depilated using a thioglycolic acid-based cream (Tosowoong Co., Ltd., Seoul, Republic of Korea), rinsed with saline, and followed by application of 100 mg of Biostir^®^ AD. For subsequent inductions, 150 µL of 4% SDS was applied and dried for 2–3 h prior to Biostir^®^ AD reapplication [18].

Dexamethasone (G3) and BCL-2 (G4–G6) treatments were initiated in week 3 and administered daily for 4 weeks. The BCL-2 formulation was freshly prepared each day by suspending the appropriate amount of powder in sterile saline to yield final concentrations of 10^6^, 10^7^, or 10^8^ CFU per 200 µL. A 200 µL aliquot of each prepared suspension was administered via oral gavage using a gastric needle.

All experimental procedures were conducted in accordance with the ethical guidelines for animal research and were approved by the Animal Experimental Ethics Committee of HLB Co., Ltd. (BIOSTEP IACUC 24-HB-0282).

### 2.4. Body and Spleen Weight and Clinical Signs Assessment

Body weight was measured weekly to monitor general health and treatment-related changes. At week 6 (day 28 of treatment), mice were euthanized, and spleens were harvested and weighed. The severity of dermatological conditions was evaluated weekly using a standardized scoring system based on four criteria: erythema/hemorrhage, edema, excoriation/erosion, and scaling/dryness [19]. The sum of the four scores was defined as the clinical severity score for each animal.

### 2.5. Flow Cytometry Analysis

Peripheral blood was collected from the tail vein into EDTA-coated tubes on week 6 (day 28 of treatment), gently mixed to prevent clotting, and processed within 2 h [20,21]. To assess inflammation by cell type, leukocyte populations, including total leukocytes, lymphocytes, neutrophils, and eosinophils, were quantified using an automated hematology analyzer (ADVIA 2120, Siemens, Washington, WA, USA), following the manufacturer’s protocol [22]. Leukocyte subtypes were automatically characterized based on cell size, granularity, and nuclear complexity.

Splenocyte-based analysis was conducted to evaluate systemic immunomodulatory effects, as described in previous studies [23]. Briefly, spleens were homogenized into single-cell suspensions using a sterile 40 µm cell strainer. Red blood cells were lysed using ACK lysis buffer (A1049201, Gibco, Waltham, MA, USA), and viable splenocytes were counted using a hemocytometer with trypan blue exclusion [24]. A total of 1 × 10^6^ splenocytes were stimulated for 4 h with phorbol myristate acetate (PMA, Thermo Scientific Chemicals, Waltham, MA, USA) and ionomycin in the presence of GolgiPlug (BD Biosciences, San Diego, CA, USA).

Surface staining was performed on ice using anti-CD4-FITC (clone: RM4-5, BioLegend, San Diego, CA, USA), followed by fixation, permeabilization, and intracellular staining with anti-IFN-γ-PE (clone: XMG1.2) and anti-IL-4-APC (clone: 11B11, BioLegend) according to the manufacturer’s protocols. The isotype controls showed <0.1% positivity. Data were acquired using a flow cytometer (FACSVerse™, BD Biosciences, San Jose, CA, USA) and analyzed using FlowJo software (FlowJo, Ashland, OR, USA) [24]. Gating strategy and representative dot plots for Th1 (CD4^+^IFN-γ^+^) and Th2 (CD4^+^IL-4^+^) cell populations are presented in Supplementary Figure S1.

### 2.6. Inflammatory Cytokine Gene Expression Analysis

Full-thickness dorsal skin lesions (~1 cm^2^) were excised, homogenized in TRIzol reagent (Thermo Fisher Scientific, Waltham, MA, USA), and phase-separated with chloroform [19]. RNA was precipitated using isopropanol, washed with 75% ethanol, and resuspended in RNase-free water. The concentration and purity of the RNA were determined using a NanoDrop spectrophotometer (2000/2000c, Thermo Fisher Scientific, Waltham, MA, USA). Reverse transcription was performed using 1 µg of total RNA, with a cDNA synthesis kit (Takara Bio, San Jose, CA, USA). qRT-PCR was conducted using SYBR Green Master Mix (Bio-Rad, Irvine, CA, USA) on a real-time PCR system (CFX96, Bio-Rad Laboratories, Hercules, CA, USA) [19]. Cytokines and chemokines gene expression was quantified using previously validated primer sequences (Supplementary Table S1). Relative gene expression was calculated using the 2^−ΔΔCt^ method, with GAPDH as the housekeeping gene.

### 2.7. Evaluation of Histopathological Lesions

Full-thickness dorsal skin (1 cm^2^) was excised, rinsed with phosphate-buffered saline (PBS), and immediately fixed in 10% neutral-buffered formalin. Tissues were dehydrated, embedded in paraffin, and sectioned at 4 μm thickness using a microtome [25]. Histological staining was performed using a commercial H&E Stain Kit (ab245880; Abcam, Cambridge, UK) according to the manufacturer’s instructions. Briefly, the sections were hydrated, stained with Mayer’s hematoxylin for 5 min, rinsed, treated with a bluing reagent for 10–15 s, and counterstained with eosin Y solution for 2–3 min. The stained sections were dehydrated in absolute ethanol and mounted with synthetic resin. Histopathological features were observed under a light microscope (CKX41, Olympus, Tokyo, Japan) at 400× magnification. Quantitative analysis of epidermal thickness (as an indicator of hypertrophy), hyperkeratosis, and inflammatory cell infiltration was performed using ImageJ software (version 1.53t; NIH, Bethesda, MD, USA) [26]. Semi-quantitative pathologic-scored lesions, including epidermal hypertrophy, hyperkeratosis, and dermal inflammation, were assessed for group comparison using a scoring system adapted from Taniguchi et al. [27], as described in Table 1. In addition, toluidine blue staining was used to identify mast cells, with inflammatory cell infiltration and mast cell density quantified in high-power fields (400×). Hyperplasia and parakeratosis were not included in the scope of histological assessment in this study.

### 2.8. Statistical Analysis

Data are presented as mean ± standard error of the mean (SEM). Statistical analyses were conducted using GraphPad Prism 9.0 (GraphPad Software, San Diego, CA, USA). Group differences were evaluated by one-way analysis of variance (ANOVA) followed by Tukey’s post hoc test for multiple comparisons. Non-parametric data were analyzed using the Kruskal–Wallis test. Statistical significance was set at *p* < 0.05. All in vitro experiments and in vivo analyses in the AD model were performed in triplicate. All statistical methods applied were based on standard procedures described by Motulsky [28].

## 3. Results

### 3.1. Synergistic Anti-Allergic Effects of LRCC and RAPO in RBL-2H3 Cells

**Figure 1:**
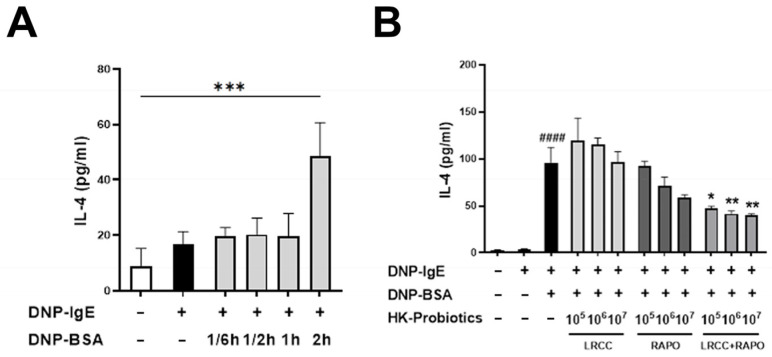
IL-4 secretion levels in DNP-IgE/DNP-BSA-stimulated RBL-2H3 cells treated with LP-5264, BL-RAPO, or BCL-2. (**A**) IL-4 induction in RBL-2H3 cells by DNP-IgE/DNP-BSA stimulation; (**B**) IL-4 secretion after treatment with LP-5264, BL-RAPO, or BCL-2 at different concentrations. ^####^ Indicates statistical significance between untreated and DNP-IgE/DNP-BSA-treated groups. Statistical significance between groups is indicated by underlined text and asterisks: *p* < 0.05 (*), *p* < 0.01 (**), *p* < 0.001 (***).

In IgE-sensitized RBL-2H3 cells, treatment with LP-5264, BL-RAPO, or BCL-2 at 10^7^ CFU/mL significantly decreased IL-4 secretion compared to DNP-BSA treatment alone (Figure 1). At this concentration, the IL-4 levels were 96.43 ± 11.36, 58.60 ± 3.14, and 40.37 ± 1.24 pg/mL for LP-5264, BL-RAPO, and BCL-2, respectively. Among the treated groups, the BCL-2 group showed the highest reduction in IL-4 secretion.

### 3.2. Evaluation of Body and Spleen Weight and Clinical Signs

No mortality was observed in any group during the experimental period. The changes in body weight during the 4-week animal experiment are summarized in Table 2. While most groups showed no significant differences in weight changes over time, the G4 group exhibited a significant increase after day 14. In contrast, G3 showed a significant decrease from 24.6 ± 0.7 g at baseline to 23.0 ± 0.5 g at day 28, which was significantly lower than the G2 group at the same time point (*p* < 0.05).

In addition, spleen weight analysis revealed increased spleen weight (0.2 ± 0.0 g, 0.7 ± 0.1% of body weight) in the G2 group compared with that of the G1 group (*p* < 0.001). In contrast, the G3 group showed the lowest spleen weight (0.1 ± 0.0 g, 0.2 ± 0.0% of body weight), showing a significant decrease compared to the G2 group (*p* < 0.05). The BCL-2-treated groups (G4, G5, and G6) showed spleen weights of 0.1 ± 0.0 g and relative spleen weights of 0.5 ± 0.0%, with no significant differences compared to G2.

**Figure 2:**
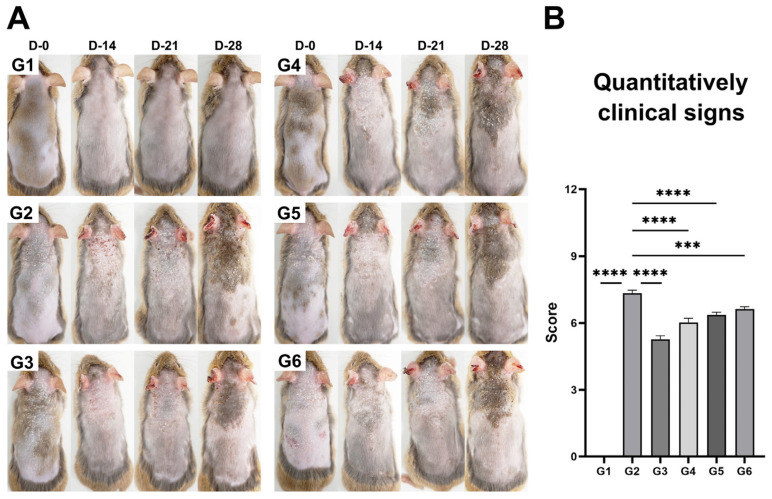
Effects of the LP-5264 and BL-RAPO on clinical signs in an atopic dermatitis mouse model. (**A**) Representative clinical signs observed across all groups; (**B**) quantitatively assessed clinical signs. G1: Control without AD; G2: AD-induced; G3: AD-induced + dexamethasone; G4: AD-induced + BCL-2 (1 × 10^6^ CFU); G5: AD-induced + BCL-2 (1 × 10^7^ CFU); G6: AD-induced + BCL-2 (1 × 10^8^ CFU). Statistical significance between groups is indicated by underlined text and asterisks: *p* < 0.001 (***), *p* < 0.0001 (****) compared to the G2.

Clinical signs of dermatitis, such as erythema and edema, were observed in all AD-induced groups (G2–G6, Figure 2A). In G2, these worsened until day 21, whereas G3, G4, and G5 showed improvements after day 14. By day 28, partial signs remained in G5 and G6 groups. In addition, clinical signs were quantitatively assessed on day 28, with severity scores highest in G2 (7.3 ± 0.1) and lowest in G3 (5.3 ± 0.2), while intermediate scores were observed in G4 (6.0 ± 0.2), G5 (6.4 ± 0.1), and G6 (6.6 ± 0.1), as shown in Figure 2B.

### 3.3. Peripheral Leukocyte Profiles and Splenic Th1/Th2 Ratio

**Figure 3:**
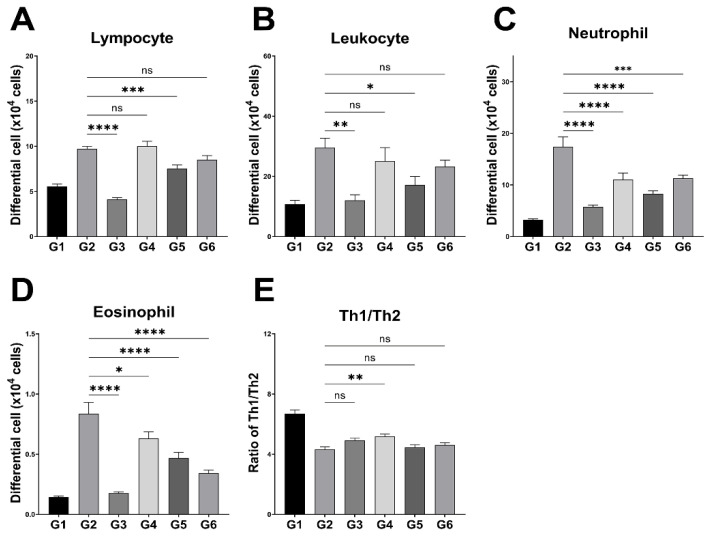
Effects of the LP-5264 and BL-RAPO on peripheral leukocyte profiles and splenic Th1/Th2 ratio in atopic dermatitis mouse model. (**A**) Lymphocyte; (**B**) leukocyte; (**C**) neutrophil; (**D**) eosinophil; (**E**) Th1/Th2 ratio in splenic CD4^+^ T cells. G1: Control without AD; G2: AD-induced; G3: AD-induced + dexamethasone; G4: AD-induced + BCL-2 (1 × 10^6^ CFU); G5: AD-induced + BCL-2 (1 × 10^7^ CFU); G6: AD-induced + BCL-2 (1 × 10^8^ CFU). Statistical significance between groups is indicated by underlined text and asterisks: *p* < 0.05 (*), *p* < 0.01 (**), *p* < 0.001 (***), *p* < 0.0001 (****). ^ns^ Indicates no statistical difference between the groups.

Leukocyte populations in peripheral blood and the splenic Th1/Th2 ratio are shown in Figure 3. The G4 group exhibited the highest lymphocyte count (10.0 ± 0.5 × 10^4^ cells), comparable to G2, whereas G3 showed the lowest count. The G5 and G6 groups presented intermediate levels, with lymphocyte counts in G3 and G5 significantly lower than those in G2. Neutrophil counts were highest in G2 and significantly decreased in G3, G4, G5, and G6. A similar trend was observed in eosinophils, with the highest count in G2 and significant reductions in all treatment groups. Flow cytometry analysis showed the highest Th1/Th2 ratio in G1. Among AD-induced groups, G2 exhibited the lowest ratio (4.3 ± 0.2), while G4 showed the highest (5.2 ± 0.2). No significant differences were observed in the ratios of G3, G5, and G6 compared to G2.

### 3.4. Inflammatory Cytokine mRNA Expression

**Figure 4:**
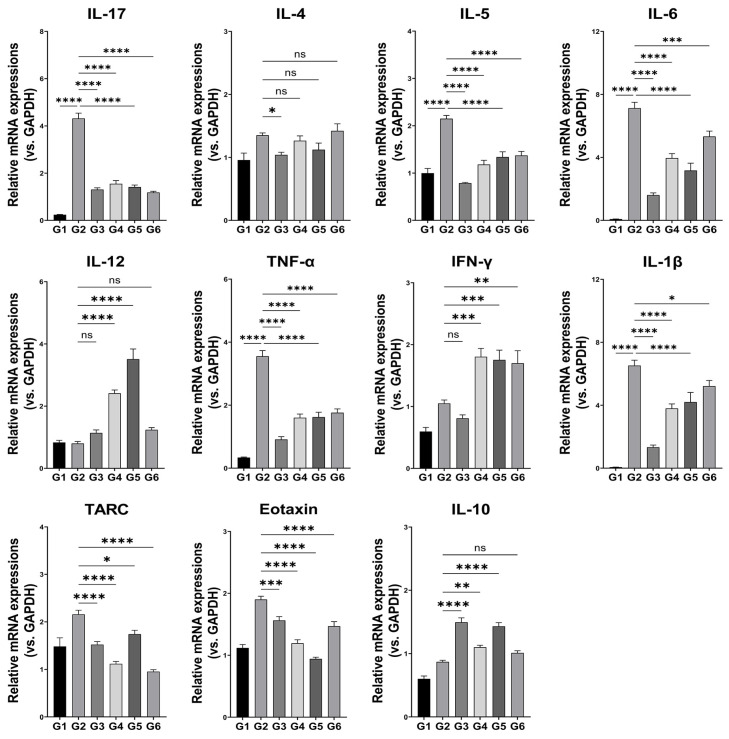
Effects of the LP-5264 and BL-RAPO on relative mRNA expression of inflammatory cytokines in atopic dermatitis mouse model. G1: Control without AD; G2: AD-induced; G3: AD-induced + dexamethasone; G4: AD-induced + BCL-2 (1 × 10^6^ CFU); G5: AD-induced + BCL-2 (1 × 10^7^ CFU); G6: AD-induced + BCL-2 (1 × 10^8^ CFU). Statistical significance between groups is indicated by underlined text and asterisks: *p* < 0.05 (*), *p* < 0.01 (**), *p* < 0.001 (***), *p* < 0.0001 (****). ^ns^ Indicates no statistical difference between the groups.

Inflammatory cytokine expression data are shown in Figure 4. The expression of IL-17, IL-4, IL-5, IL-6, TNF-α, IL-1β, TARC, and eotaxin were significantly decreased in the G3 group compared to those in the G2 group. In contrast, IFN-γ expression was significantly increased in the G3 group relative to the G2 group. Among the BCL-2-treated groups, both G4 and G5 exhibited significantly lower expression levels of IL-17, IL-5, IL-6, TNF-α, IL-1β, TARC, and eotaxin compared to the G2 group. Although IL-4 expression was reduced, the difference was not statistically significant. In contrast, IL-12 and IFN-γ levels were significantly increased relative to the G2 group. In the G6 group, the expression of IL-17, IL-5, IL-6, TNF-α, IL-1β, TARC, and eotaxin were significantly decreased compared to that of the G2 group. The anti-inflammatory marker IL-10 was significantly elevated in G3, G4, and G5 compared to G2, with the highest expression observed in G3 (1.6 ± 0.2), followed by G5 (1.5 ± 0.2) and G4 (1.2 ± 0.2).

### 3.5. Histological Analysis

**Figure 5:**
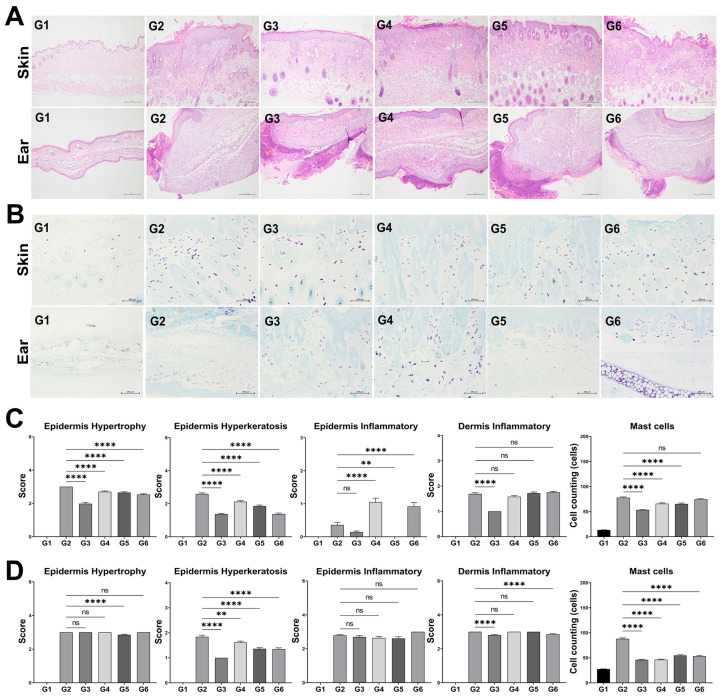
Effects of the LP-5264 and BL-RAPO on histology and mast cell counts in atopic dermatitis mouse model. (**A**) Representative histological images; (**B**) Representative images of toluidine blue-stained skin sections (×200); (**C**) semi-quantitative scores in skin tissue; (**D**) semi-quantitative scores in ear tissue. G1: Control without AD; G2: AD-induced; G3: AD-induced + dexamethasone; G4: AD-induced + BCL-2 (1 × 10^6^ CFU); G5: AD-induced + BCL-2 (1 × 10^7^ CFU); G6: AD-induced + BCL-2 (1 × 10^8^ CFU). Statistical significance between groups is indicated by underlined text and asterisks: *p* < 0.01 (**), *p* < 0.0001 (****). ^ns^ Indicates no statistical difference between the groups.

Representative histological images of the skin and ear tissues are shown in Figure 5A. Notable reductions in epidermal thickening, hyperkeratosis, and inflammatory cell infiltration were observed in the G3, G5, and G6 groups, with G3 exhibiting the most pronounced improvement. Histopathological changes in skin and ear tissues were semi-quantitatively evaluated based on pathologic-scored lesions (Figure 5C,D). In the skin (Figure 5C), G2 exhibited the most severe hypertrophy, hyperkeratosis, and dermal/epidermal inflammation. G3 showed significantly reduced hypertrophy (2.0 ± 0.0), hyperkeratosis (1.4 ± 0.0), and epidermal inflammation (0.1 ± 0.0) compared to G2. Among BCL-2 groups, G6 showed the lowest hypertrophy (2.5 ± 0.1) and hyperkeratosis (1.4 ± 0.1), while G5 had the lowest epidermal inflammation (0.2 ± 0.1). Dermal inflammation was the lowest in G3 and highest in G6. Mast cell infiltration in skin tissue was the lowest in the G3 group (53.3 ± 1.0 cells), followed by G4 (65.7 ± 1.7 cells) and G5 (65.3 ± 1.9 cells), all significantly reduced compared with the G2 group.

In the ear tissue (Figure 5D), hyperkeratosis scores were significantly lower in all groups compared to the G2 group. For hypertrophy, only the G5 group showed a significant reduction. Dermal inflammation was significantly reduced in the G3 and G6 groups, while epidermal inflammation showed no significant differences among the AD-induced groups. Mast cell counts in the ear tissue were lowest in the G3 (46.1 ± 0.9 cells) and G4 (46.4 ± 0.9 cells) groups.

## 4. Discussion

AD is a chronic inflammatory skin condition characterized by excessive activation of Th2 immune responses, leading to IgE-mediated mast cell degranulation and increased secretion of pro-inflammatory cytokines, including IL-4, IL-5, and IL-13 [1,2,3]. This study aimed to evaluate the immunomodulatory effects of the BCL-2 probiotic mixture by analyzing IL-4 secretion in vitro and assessing various immune and histopathological parameters in an AD-induced mouse model.

Inflammatory cytokines secreted by immune cells, including RBL-2H3 cells, play critical roles in IgE-mediated hypersensitivity and immune regulation. IL-4, a key Th2 cytokine, facilitates IgE production and promotes mast cell and eosinophil activation [16]. In the present study, treatment with either individual strains or the BCL-2 mixture significantly suppressed IL-4 secretion, indicating immunomodulatory and anti-inflammatory properties. Notably, the BCL-2 combination exerted a higher inhibitory effect than the single strains, suggesting a potential synergistic interaction [29]. These findings align with previous reports highlighting enhanced efficacy of multi-strain probiotics. While the reduction in IL-4 supports mast cell inhibition by BCL-2, the mechanistic link to the modulation of T-cell responses observed in vivo remains unclear. A potential mechanism is that probiotic-derived components may interact with pattern recognition receptors (PRRs), such as Toll-like receptors (TLR2 and TLR9), on mast cells, which could in turn influence cytokine secretion and modulate the local immune environment [13]. Further investigation is required to clarify the in vivo mechanisms underlying mast cell regulation and subsequent T-cell modulation by BCL-2.

After 6 weeks, significant weight loss was observed in the dexamethasone-treated group among AD-induced mice, whereas no weight loss occurred in the BCL-2-treated groups. Dexamethasone is known to induce metabolic alterations, including weight loss and muscle wasting, due to changes in the basal metabolic rate [7]. Although it provides immunomodulatory benefits, its prolonged use warrants caution due to these adverse effects. In contrast, BCL-2 treatment did not result in weight reduction, indicating a potentially safer profile in terms of metabolic side effects. However, because body and spleen weight alone may not comprehensively reflect systemic or organ-specific toxicity, the absence of biochemical enzyme analyses and histopathological evaluations of major organs such as the liver represents a limitation of the present study. Future studies should therefore incorporate more comprehensive toxicity assessments to better define the safety profile of BCL-2.

AD clinical signs, including erythema and edema, result from immune dysregulation and skin barrier damage [30]. Chronic inflammation reduces barrier proteins (e.g., filaggrin and loricrin), increases transepidermal water loss, and enhances antigen penetration, exacerbating the disease [30]. Stefanovic and Irvine described how deficiencies in skin barrier proteins exacerbate AD clinical signs, including erythema and edema, due to increased transepidermal water loss [30]. Similarly, Kwon et al. reported that oral administration of *L. sakei* WIKIM30 significantly alleviated erythema and edema in NC/Nga mice, resulting in a marked reduction in skin lesion scores [31]. In line with these findings, BCL-2 treatment in the present study significantly alleviated erythema and edema, supporting previous evidence that probiotics can improve skin barrier function and mitigate AD clinical signs [30,31]. No dose-dependent differences were observed, suggesting that immune modulation may reach a saturation point, possibly due to receptor desensitization or limitations in downstream mechanisms requiring further investigation.

AD pathology is primarily driven by an imbalance in inflammatory cytokines and immune cell populations. While draining lymph nodes mediate local T-cell responses, the spleen reflects systemic immunomodulation, which has previously been shown to be modulated by probiotic treatment in murine AD models [31]. Th2-mediated responses promote IgE-driven inflammation and overexpression of neutrophils, eosinophils, and mast cells. Neutrophils exacerbate acute inflammation, while eosinophils contribute to barrier disruption [1,5]. BCL-2 treatment reduced neutrophil and eosinophil counts, suggesting attenuated immune activation. Some BCL-2 groups also showed an increased Th1/Th2 ratio. Similar effects have been reported in previous studies. Kwon et al. [31] demonstrated that oral administration of *L. sakei* WIKIM30 in NC/Nga mice increased Th1 cytokines, including IFN-γ, while reducing IL-4, thereby restoring the Th1/Th2 balance and ameliorating AD-like skin lesions. Likewise, Chen et al. [32] showed that heat-killed *L. salivarius* MP01 and *L. johnsonii* MP02 elevated IFN-γ and suppressed IL-4 and IL-13 levels, resulting in a higher Th1/Th2 ratio in HDM-induced AD mice. These findings are consistent with the present results, suggesting that modulation of the Th1/Th2 balance may be a key mechanism underlying the anti-atopic effects of BCL-2. However, as CD4^+^ IFN-γ^+^ (Th1) cells comprise a small proportion of CD4^+^ T cells, these changes may be of limited biological relevance. Further studies are required to clarify the immunomodulatory mechanisms of BCL-2, particularly regarding Th1/Th2 regulation and symptom improvement.

Among the BCL-2-treated groups, G4 and G5 significantly decreased the expression of key pro-inflammatory cytokines, including IL-5 and IL-17, which facilitate eosinophil activation and sustain inflammation by promoting the recruitment of inflammatory cells to affected tissues [3]. Conversely, IL-10, a major anti-inflammatory cytokine that suppresses excessive immune cell activation, was increased, consistent with previous reports on probiotic-mediated immune modulation [12,29]. Kim et al. demonstrated that a probiotic mixture elevated IL-10 expression while reducing Th2 cytokines, thereby restoring immune balance and alleviating AD in mice [12], suggesting that BCL-2 may exert similar effects through IL-10–mediated immune regulation, thus suggesting that BCL-2 may contribute to the improvement of AD. Although IL-4 expression was significantly suppressed in vitro, its serum levels in the AD mouse model exhibited only a non-significant decreasing trend. This discrepancy likely reflects the complexity of immune regulation in vivo, where systemic cytokine levels may not accurately reflect localized immune activity [21,33]. IL-4 contributes to AD pathogenesis by activating mast cells and eosinophils and by inducing chemokines such as TARC and eotaxin [1,3,34]. These downstream mediators play pivotal roles in recruiting Th2 cells and eosinophils to inflamed skin lesions, thereby amplifying allergic inflammation [34]. Given the multifactorial pathophysiology of AD, which includes Th2-polarized immune responses, epithelial-derived cytokines such as thymic stromal lymphopoietin (TSLP), and chemokine-mediated immune cell recruitment, comprehensive understanding of BCL-2–mediated modulation requires integrative analysis of these interacting pathways. Therefore, in addition to IL-4, it is essential to evaluate the coordinated regulation of its downstream markers, including TARC and eotaxin, as well as other key mediators involved in Th2-dominant inflammation and epithelial–immune crosstalk [35,36]. Future studies should incorporate tissue-specific cytokine profiling and mechanistic investigations in both intestinal and cutaneous compartments to delineate the immunological mechanisms underlying BCL-2 efficacy.

In addition to clinical signs such as erythema, edema, and increased spleen weight, histopathological features—including epidermal hypertrophy, hyperkeratosis, dermal inflammatory cell infiltration, and mast cell accumulation—represent the major postmortem pathological changes associated with AD. Among them, epidermal thickening results from chronic inflammation, which also amplifies immune responses and tissue damage [31,32]. In the present study, these pathological changes were alleviated by BCL-2 treatment, indicating anti-inflammatory and immunomodulatory effects. A similar reduction in epidermal thickness was observed by Kwon et al., who reported that *L*. *sakei* WIKIM30 administration improved AD-like skin lesions in NC/Nga mice by suppressing skin inflammation and immune cell infiltration [31]. In addition, mast cell infiltration—known to drive allergic responses via histamine, leukotriene, and prostaglandin release [37]—was significantly reduced in the BCL-2 groups. These findings support that BCL-2 may attenuate inflammation by modulating mast cell activity, consistent with previous reports on probiotic-mediated immune regulation. Eom et al. demonstrated that *P. pentosaceus* KF159 alleviated house dust mite-induced atopic dermatitis in mice by suppressing mast cell degranulation and promoting IL-10 production, highlighting mast cells as key targets in probiotic-based anti-inflammatory strategies [38]. However, the keratohyalin granule layer, which is known to be associated with epidermal differentiation and skin barrier function in AD [39], was not independently assessed in this study. Further evaluation of this component may clarify the barrier-restorative effects of probiotics.

BCL-2 exerted significant immunomodulatory effects by regulating immune cell activity, inflammatory cytokine expression, and mast cell responses, contributing to the alleviation of AD clinical signs, such as erythema and edema. The efficacy varied by dose, with G4 (10^6^ CFU) and G5 (10^7^ CFU) showing pronounced anti-inflammatory and histological improvements, while G6 (10^8^ CFU) exhibited weaker effects. This pattern may reflect dose-dependent immunological responses, as excessive probiotic administration has been linked to aberrant immune activation or diminished efficacy, particularly in vulnerable hosts [16,23]. These findings suggest that intermediate doses of BCL-2, especially 10^7^ CFU, may offer optimal therapeutic benefit while minimizing potential risks.

However, this study has certain limitations. The murine model may not fully reflect human AD pathology, limiting its clinical translation. Moreover, BCL-2 treatment did not significantly elevate IL-4 expression, indicating a limited effect on the Th2-mediated response. Although BCL-2 inhibits mast cell activity in vitro, its in vivo link to T-cell modulation remains unclear. Future studies should include human trials to assess efficacy and safety, as well as to investigate BCL-2’s impact on the gut microbiota and its role in immune regulation.

## 5. Conclusions

We evaluated the effects of the multi-probiotic strain formulation BCL-2, composed of LP-5264 and BL-RAPO, on alleviating AD clinical signs. In vitro, BCL-2 significantly reduced IL-4 secretion, suggesting suppression of Th2 responses. In a murine model of AD, BCL-2 alleviated clinical signs, modulated immune cell activity, decreased pro-inflammatory cytokine levels and mast cell infiltration, and increased IL-10 levels. Additionally, BCL-2 showed a favorable safety profile, supporting its potential as a safe and effective therapeutic candidate for AD management.

## Figures and Tables

**Table 1:** Criteria for semi-quantitative pathologic scoring of histological skin lesions in the murine atopic dermatitis model.

Parameter	Criteria Description	Score
Hypertrophy	No hypertrophy	0
	Mild hypertrophy (slight thickening of the epidermis)	1
	Moderate hypertrophy (increased epidermal thickness)	2
	Severe hypertrophy (marked thickening of the epidermis)	3
Hyperkeratosis	No hyperkeratosis	0
	Mild hyperkeratosis (minimal keratin layer)	1
	Moderate hyperkeratosis (evident keratin layer)	2
	Severe hyperkeratosis (thickened, compact keratin layer)	3
Inflammation	No inflammatory cell infiltration	0
	Mild infiltration (few inflammatory cells in the dermis)	1
	Moderate infiltration (scattered inflammatory cells)	2
	Severe infiltration (dense aggregation of inflammatory cells)	3

**Table 2:** Effects of the BCL-2 on body weight and organ weights.

Animal Groups	Body Weight/Experimental Duration (g/Days)					Organ Weight/Day28	
	0	7	14	21	28	Spleen (g)	% (vs. BW)
G1	24.0 ± 0.4	24.8 ± 0.4	25.3 ± 0.4	25.9 ± 0.2	26.3 ± 0.3	0.1 ± 0.0	0.3 ± 0.0
G2	23.7 ± 0.3	24.0 ± 0.5	24.2 ± 0.4	24.5 ± 0.4	24.9 ± 0.5	0.2 ± 0.0 ***	0.7 ± 0.1 ***
G3	24.6 ± 0.7	23.4 ± 0.7	23.1 ± 0.5 **	23.0 ± 0.5 ***	23.0 ± 0.5 ***^,#^	0.1 ± 0.0 ^#^	0.2 ± 0.0 ^#^
G4	22.7 ± 0.5	23.1 ± 0.4	23.6 ± 0.3 *	23.9 ± 0.4 **	24.3 ± 0.5 **	0.1 ± 0.0	0.5 ± 0.0
G5	24.8 ± 0.6	25.0 ± 0.7	25.0 ± 0.6	25.1 ± 0.6	25.3 ± 0.5	0.1 ± 0.0	0.5 ± 0.0
G6	23.8 ± 0.4	24.3 ± 0.3	24.7 ± 0.2	24.5 ± 0.3	25.4 ± 0.3	0.1 ± 0.0	0.5 ± 0.0

G1: Control without AD; G2: AD-induced; G3: AD-induced + dexamethasone; G4: AD-induced + BCL-2 (1 × 10^6^ CFU); G5: AD-induced + BCL-2 (1 × 10^7^ CFU); G6: AD-induced + BCL-2 (1 × 10^8^ CFU). Statistical significance between groups is indicated by underlined text and asterisks: *p* < 0.05 (*), *p* < 0.01 (**), *p* < 0.001 (***). ^#^ Indicates a significant difference compared with the G2.

## Data Availability

The original contributions presented in this study are included in the article and Supplementary Materials. Further inquiries can be directed to the corresponding authors.

## Supplementary Materials

The following supporting information can be downloaded at https://www.mdpi.com/article/10.3390/microorganisms13081950/s1, Table S1: Primer sequences for qRT-PCR analysis; Table S2: Classification and functional roles of cytokines in atopic dermatitis; Figure S1: Representative flow cytometry gating strategy for the identification of CD4^+^IFN-γ^+^ and CD4^+^IL4^+^ cells in splenocytes.

## Abbreviations

The following abbreviations are used in this manuscript:

AD	Atopic Dermatitis
ANOVA	Analysis of Variance
BL-RAPO	*Bifidobacterium longum* RAPO
DNP	Dinitrophenyl
DNP-BSA	Dinitrophenyl–Bovine Serum Albumin
EDTA	Ethylenediaminetetraacetic Acid
ELISA	Enzyme-Linked Immunosorbent Assay
EMEM	Eagle’s Minimum Essential Medium
FBS	Fetal Bovine Serum
LP-5264	*Lactiplantibacillus plantarum* LRCC5264
MRS	de Man, Rogosa, and Sharpe (agar/broth)
PBS	Phosphate-Buffered Saline
PMA	Phorbol 12-Myristate 13-Acetate
PRR	Pattern Recognition Receptor
SDS	Sodium Dodecyl Sulfate
SEM	Standard Error of the Mean
TLR	Toll-Like Receptor
TOS	Transgalactooligosaccharide
