# Supplementary material for: BCL-2 Multi-Strain Probiotics for Immunomodulation In Vitro and In Vivo Alleviation of Atopic Dermatitis
Source: Microorganisms. 2025 Aug 21;13(8):1950. doi: 10.3390/microorganisms13081950 (PMC12388198; doi:10.3390/microorganisms13081950)

Supplementary Figure 1. Representative flow cytometry gating strategy for the identification of CD4⁺ IFN-γ⁺ and CD4⁺ IL4⁺ cells in splenocytes.

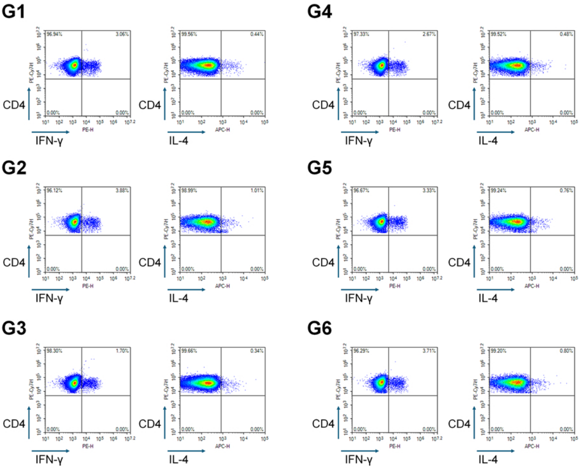

Supplement: Supplementary file 1 [file microorganisms-13-01950-s001.zip › Supplementary Figure.pdf]
