# Supplementary material for: BCL-2 Multi-Strain Probiotics for Immunomodulation In Vitro and In Vivo Alleviation of Atopic Dermatitis
Source: Microorganisms. 2025 Aug 21;13(8):1950. doi: 10.3390/microorganisms13081950 (PMC12388198; doi:10.3390/microorganisms13081950)
Supplement: Supplementary file 1 [file microorganisms-13-01950-s001.zip › Supplementary Table.pdf]

Supplementary Table S1. Primer sequences for qRT-PCR analysis.

Gene	Primer direction	Primer sequences
GAPDH	Forward	AAATGGTGAAGGTCGGTGTGAAC
	Reverse	CCACAATCTCCACTTTGCCACTG
IFN-γ	Forward	TCAAGTGGCATAGATGTGGAAGAA
	Reverse	TGGCTCTGCAGGATTTTCATG
IL-4	Forward	ACAGGAGAAGGGACGCCAT
	Reverse	GAAGCCCTACAGACGAGCTCA
IL-5	Forward	CAAAAAGAGAAGTGTGGCGAGG
	Reverse	TAGATAGGAGCAGGAAGCCCCG
IL-6	Forward	GAGGATACCACTCCCAACAGACC
	Reverse	AAGTGCATCATCGTTGTTCATACA
IL-10	Forward	TGAATTCCCTGGGTGAGAAGCTGA
	Reverse	TGGCCTTGTAGACACCTTGGTCTT
IL-12	Forward	TGAACTGGCGTTGGAAGC
	Reverse	GCGGGTCTGGTTTGATGA
IL-13	Forward	CAGCCTCCCCGATACCAAAA
	Reverse	TCCTCATTAGAAGGGGCCGT
IL-17	Forward	AAGGCAGCAGCGATCATCC
	Reverse	GGAACGGTTGAGGAGTCTGAG
IL-1β	Forward	TGAAGCAGCTATGGCAACTG
	Reverse	GGGTCCGTCAACTTCAAAGA
TNF-α	Forward	CGTCGTAGCAAACCACCAAG
	Reverse	TTGAAGAGAACCTGGGAGTAGACA
TARC	Forward	GGGATGCCATCGTGTTTCTG
	Reverse	CTGTCCAGGGCAAGCTCATCTGTGC
Eotaxin	Forward	GGCTGACCTCAAACTCACAGAAA
	Reverse	ACATTCTGGCTTGGCATGGT

Supplementary Table S2. Classification and functional roles of cytokines in atopic dermatitis

Cytokine	Classification	Role in AD Pathogenesis
IL-4 [1]	Th2	Induces IgE class switching in B cells; promotes mast cell activation and Th2 differentiation
IL-5 [1]	Th2	Enhances eosinophil activation and survival; contributes to chronic inflammation
IL-6 [3]	Mixed (Th2/Th17)	Amplifies inflammatory response; promotes Th17 differentiation and acute phase response
IL-10 [2]	Treg	Anti-inflammatory; suppresses Th1/Th2 cytokine production and mast cell activation
IL-12 [1]	Th1	Promotes Th1 differentiation and IFN-γ production; counter-regulates Th2 responses
IL-17 [1]	Th17	Induces neutrophilic inflammation; disrupts epithelial barrier by downregulating tight junctions
IL-1β [2]	Pro-inflammatory	Induces expression of adhesion molecules and chemokines; amplifies inflammatory cascade
TNF-α [1]	Pro-inflammatory	Enhances expression of adhesion molecules; promotes skin inflammation and apoptosis
IFN-γ [1]	Th1	Suppresses Th2-mediated inflammation; enhances skin barrier proteins
TARC/CCL17 [2]	Th2-related chemokine	Recruits $CCR4^+$ Th2 cells into inflamed skin; correlates with AD severity
Eotaxin/CCL11 [2]	Eosinophil chemokine	Attracts eosinophils into dermis; sustains allergic inflammation

[1] Brunner, P.M.; Guttman-Yassky, E.; Leung, D.Y.M. The immunology of atopic dermatitis and its reversibility with broad-spectrum and targeted therapies. J. Allergy Clin. Immunol. 2017, 139, 65–76.
[2] Homey, B.; Steinhoff, M.; Ruzicka, T.; Leung, D.Y.M. Cytokines and chemokines orchestrate atopic skin inflammation. J. Allergy Clin. Immunol. 2006, 118, 178–189.
[3] Zhao, S.S.; Yiu, Z.Z.N. Genetically proxied IL-6 receptor inhibition is associated with increased risk of atopic dermatitis. J. Invest. Dermatol. 2024, 154, 666–669.
